# Impact of Eccentric Exercise Interventions with Small and Large Ranges of Motion on Rat Skeletal Muscle Tissue and Muscle Force Production

**DOI:** 10.3390/ijms25168978

**Published:** 2024-08-18

**Authors:** Ryoya Oga, Koki Nakagawa, Yi-Chen Chen, Yoshihiro Nita, Hiroyuki Tamaki

**Affiliations:** Department of Sports and Life Science, National Institute of Fitness and Sports in Kanoya, 1 Shiromizu, Kanoya 891-2393, Japan; m247001@sky.nifs-k.ac.jp (R.O.); m227005@sky.nifs-k.ac.jp (K.N.); m217005@sky.nifs-k.ac.jp (Y.-C.C.); m236007@sky.nifs-k.ac.jp (Y.N.)

**Keywords:** eccentric contraction, range of motion, muscle damage, myofibers, myofibrosis, Pax7

## Abstract

Eccentric training induces greater hypertrophy while causing more muscle damage than concentric training. This study examined the effects of small-range eccentric contractions (SR-ECCs) and large-range eccentric contractions (LR-ECCs) on muscle morphology, contractility, and damage in rats. Thirty male Fischer 344 rats were divided into five groups: small-range ECC single-bout (SR-ECC_SB_, *n* = 4), large-range ECC single-bout (LR-ECC_SB_, *n* = 4), SR-ECC intervention (SR-ECC_Intv_, *n* = 7), LR-ECC intervention (LR-ECC_Intv_, *n* = 8), and control (Cont, *n* = 7). These groups underwent transcutaneous electrical stimulation involving 80 ECCs twice a week for four weeks. The results indicated that the LR-ECC_SB_ group had more Evans blue dye-positive fibers than other groups. The SR-ECC_Intv_ group showed no increase in the mean myofiber cross-sectional area. However, Pax7+ and Ki67+ cells significantly increased in both ECC_Intv_ groups compared to the Cont group, and the connective tissue area was significantly greater in the LR-ECC_Intv_ than in others. Muscle force was lower in both ECC_Intv_ groups compared to the Cont group. These findings suggest that SR-ECC intervention may induce a smaller increase in the number of fibers with a large myofiber cross-sectional area and satellite cell proliferation with less muscle damage and myofibrosis compared to LR-ECCs.

## 1. Introduction

Eccentric contraction (ECC) exercise is known to induce greater hypertrophy compared to other muscle contraction types [1]. Increasing protein synthesis and the number of myonuclei are important for muscle hypertrophy, with mechanical stress from exercise being a key factor in their stimulation [2]. ECC is characterized by its ability to generate large muscle force production due to passive forces associated with titin and the extracellular matrix (ECM) [3,4], as well as by the activation of stretch-activated channels (SACs) localized in the muscle cell membrane during muscle lengthening [5,6]. These unique features of ECC contribute to its effectiveness in promoting muscle hypertrophy. However, high-intensity ECC exercise is likely to induce various types of muscle damage, including structural changes like myofibrosis, loss of membrane integrity, inflammation, muscle pain, and muscle force reduction [7].

Reportedly, the magnitude of ECC-induced muscle damage depends on various protocol settings (intensity and volume, angular velocity, muscle elongation) and staged interventions [8]. Notably, the range of motion during ECC is critically linked to the degree of muscle damage incurred. Studies demonstrated that muscle damage induced by ECC is less severe when performed over a small range of motion compared to a large range of motion [9,10]. Hayashi et al. [11] have shown that the magnitude of mechanical hyperalgesia in muscles increases proportionally with the range of motion following ECC. Furthermore, the amount of damage resulting from eccentric exercise in tetanically activated muscle is primarily determined by the amplitude of the stretches and the range of sarcomere lengths over which the stretch occurs [12].

Muscle satellite cells (MuSCs) play a crucial role in the increase in the number of myonuclei, which is important for muscle hypertrophy. MuSCs are typically quiescent between the basement membrane and the plasma membrane, where the activation, proliferation, and differentiation of MuSCs lead to an increase in the number of myonuclei. MuSC activation is most prominent during the muscle regeneration phase that follows ECC-induced muscle injury [13,14]. However, MuSC activation has also been observed in types of muscle contractions that do not typically cause muscle damage, such as concentric contractions [15]. These findings suggest that even in the absence of muscle damage, appropriate mechanical stress applied to the muscle can increase the number of myonuclei through the activation of MuSCs. In particular, the existence of a pathway that activates MuSCs via mechanical stress independent of muscle damage is evident [16]. Furthermore, some mechanosensors associated with muscle protein synthesis are located near the plasma membrane [5,6,17]. Therefore, even in the absence of muscle damage, appropriate mechanical stress applied to skeletal muscle may increase myonuclear quantity and myoprotein synthesis, thereby effectively promoting muscle hypertrophy. Exploring the effectiveness of low-impact exercises, such as small-range ECC exercise interventions that cause less muscle damage, might be beneficial for individuals who are unable to perform high-impact activities. However, limited knowledge exists on the effects of small-range ECC exercise interventions on muscle damage, function, and morphology.

The present study examined the effectiveness of relatively small-range ECC exercise interventions on muscle damage, hypertrophy, myofibrosis, and force production profiles in rat skeletal muscle.

## 2. Results

### 2.1. Number of EBD+ Fibers and Localization of Dystrophin

**Figure 1:**
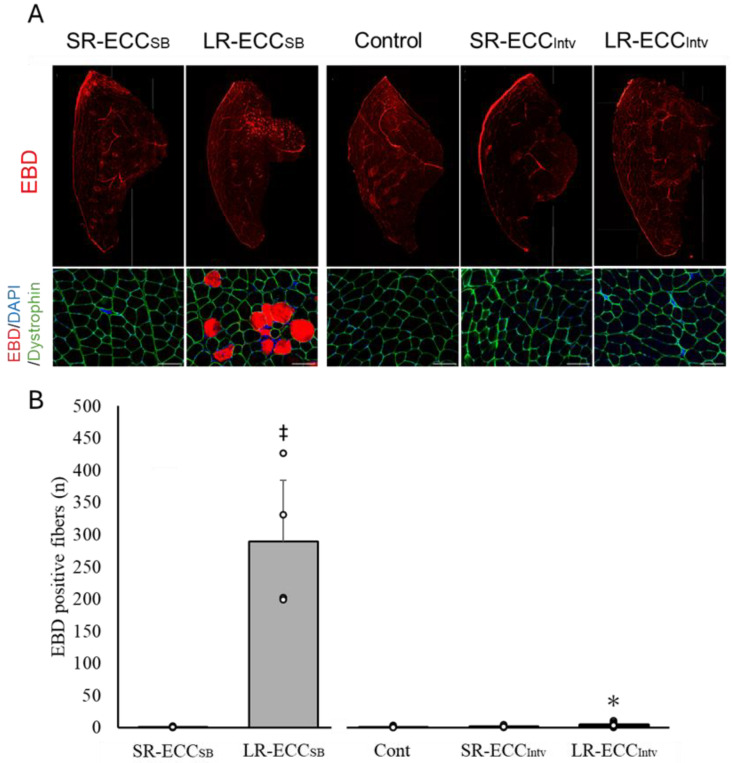
Representative cross-sectional images of tibialis anterior (TA) muscle immunostained for dystrophin (green) in the Cont, SR-ECC_Intv_, and LR-ECC_Intv_ groups (**A**), and the quantification of the mean number of EBD+ fibers (**B**). Red indicates Evans blue dye-positive fibers. Blue indicates 4′,6-Diamidino-2-phenylindole (DAPI). The scale bar represents 100 μm. * *p* < 0.05 vs. Cont, ^‡^ *p*  <  0.05 vs. SR-ECC_SB_. Values are expressed as means ± SD.

To assess the effects on muscle tissue induced by SR- and LR-ECCs, we used immunohistochemistry and histological techniques to evaluate dystrophin disruption and increased membrane permeability using EBD staining. Many dystrophin-negative and EBD-positive fibers were shown in the LR-ECC_SB_ group (Figure 1A), while fewer of these fibers were evident in the SR-ECC_SB_ group (Figure 1A). There were 289 ± 110 EBD+ fibers in the LR-ECC_SB_ group, which was significantly higher than the 1 ± 1 EBD+ fibers in the SR-ECC_SB_ group (*p* < 0.05) (Figure 1B). Conversely, the numbers of EBD+ fibers in Cont, SR-ECC_Intv_, and LR-ECC_Intv_ were 1 ± 1, 2 ± 1, and 4 ± 3, respectively (Figure 1B). The number of EBD+ fibers in the SR-ECC_Intv_ group was not significantly different from the Cont group (*p* = 0.73). Furthermore, although the number of EBD+ fibers in the LR-ECC_Intv_ group was low, it was significantly higher than that of the Cont group (*p* < 0.05).

### 2.2. Muscle Contraction Force

**Figure 2:**
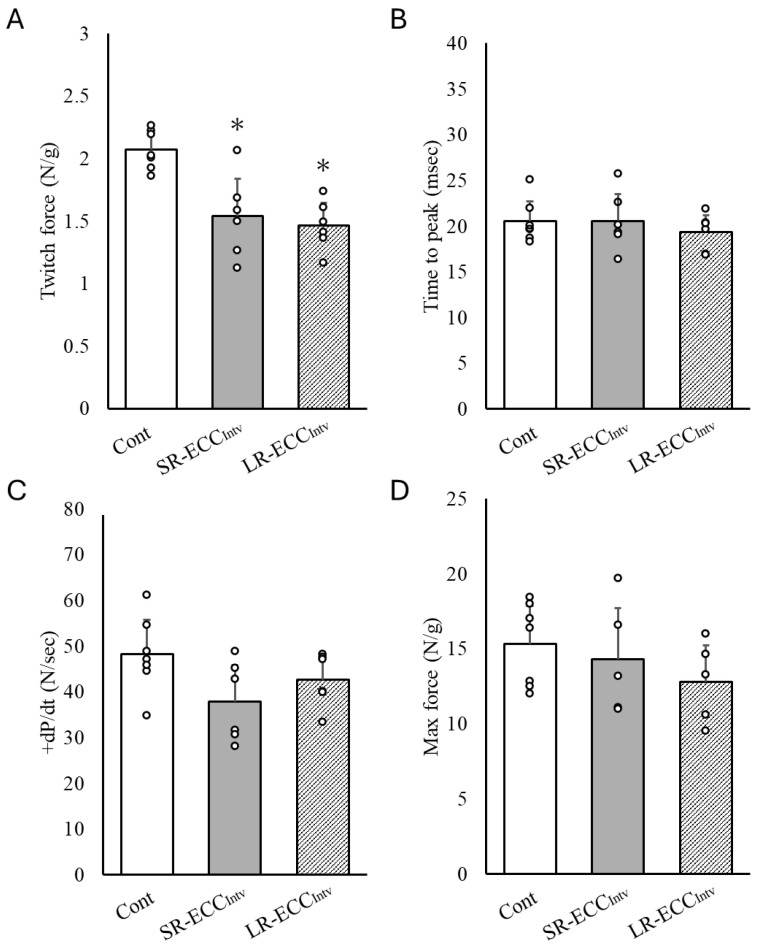
Tibialis anterior (TA) muscle twitch force (**A**), time to peak (**B**), +dP/dt (**C**), and maximal tetanic force (**D**) induced by transcutaneous electrical stimulation (ES) at an intensity of 23–25 V at 2 Hz or 125 Hz in the Cont, SR-ECC_Intv_, and LR-ECC_Intv_ groups. * *p* < 0.05 vs. Cont. Values are expressed as means ± SD.

We observed a significant decrease in twitch force in SR-ECC_Intv_ and LR-ECC_Intv_ compared to the Cont group (*p* < 0.05) (Figure 2A). However, there was no significant difference in twitch force between SR-ECC_Intv_ and LR-ECC_Intv_. Additionally, twitch +dP/dt, time to peak, and max force did not show significant differences between the groups (Figure 2B–D). However, +dP/dt was reduced by 21.4% in SR-ECC_Intv_ and 11.3% in LR-ECC_Intv_ compared to the Cont group (Figure 2C).

### 2.3. Muscle Weight, FCSA, and Connective Tissue

**Figure 3:**
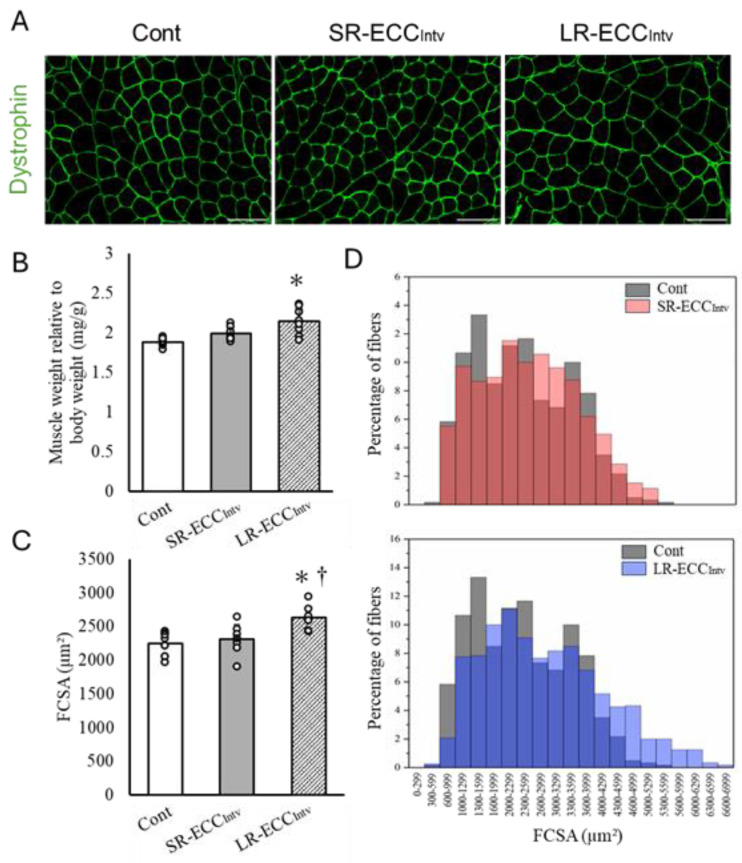
Representative cross-sectional images of tibialis anterior (TA) muscle immunostained for dystrophin (green) in the Cont, SR-ECC_Intv_, and LR-ECC_Intv_ groups (**A**), mean TA muscle weight relative to body weight (**B**), quantification of mean myofiber cross-sectional area (FCSA) (**C**), and contribution of myofiber FCSA (**D**). In figure (**D**), the dark red and dark blue colors show the overlapping FCSA distribution with control group (gray color), respectively. The scale bar represents 100 μm. * *p*  <  0.05 vs. Cont, ^†^ *p*  <  0.05 vs. SR-ECC_Intv_. Values are expressed as means  ±  SD.

**Figure 4:**
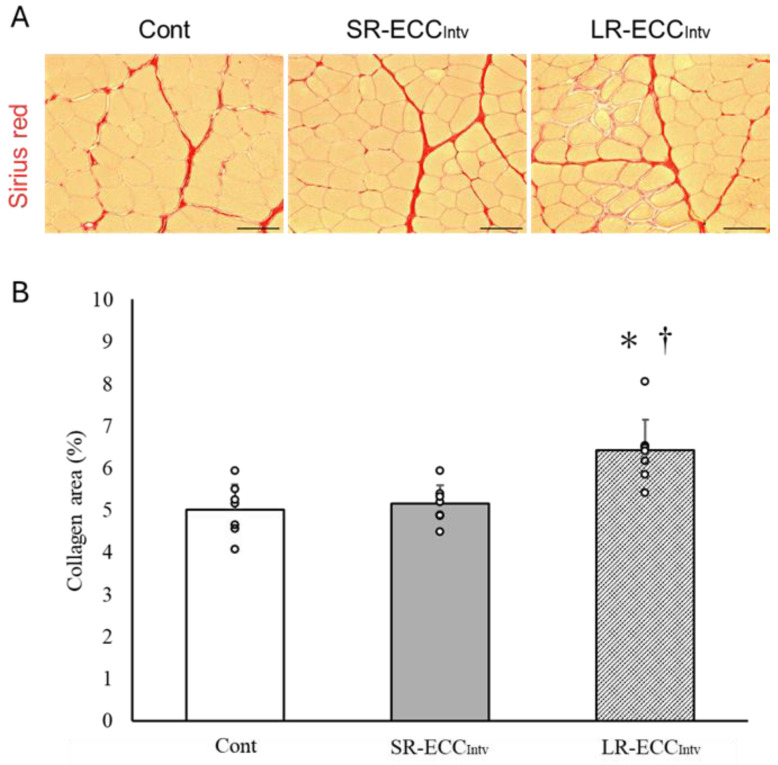
The picrosirius red-stained (red) cross-sectional images of TA muscle in the Cont, SR-ECC_Intv_, and LR-ECC_Intv_ groups (**A**), and the ratio of mean collagen area (**B**). The scale bar represents 100 μm. * *p* < 0.05 vs. Cont, ^†^ *p* < 0.05 vs. SR-ECC_Intv_. Values are expressed as means ± SD.

SR-ECC_Intv_ did not lead to discernible changes in muscle weight (*p* = 0.26), mean myofiber cross-sectional area (*p* = 0.84), or ratio of connective tissue (*p* = 0.92) compared to the Cont group (Figure 3A–C and Figure 4A,B). Conversely, LR-ECC_Intv_ resulted in significant increases in muscle weight, mean FCSA, and connective tissue ratio compared to SR-ECC_Intv_ and the Cont group (*p* < 0.05) (Figure 3A–C and Figure 4A,B). However, there was a shift in myofiber cross-sectional area distribution with SR-ECC_Intv_ compared to the Cont group (*p* < 0.05) (Figure 3D). The shift observed with LR-ECC_Intv_ was more pronounced than that observed in other groups (*p* < 0.05).

### 2.4. Increase in Satellite Cell Number and Satellite Cell Proliferation

**Figure 5:**
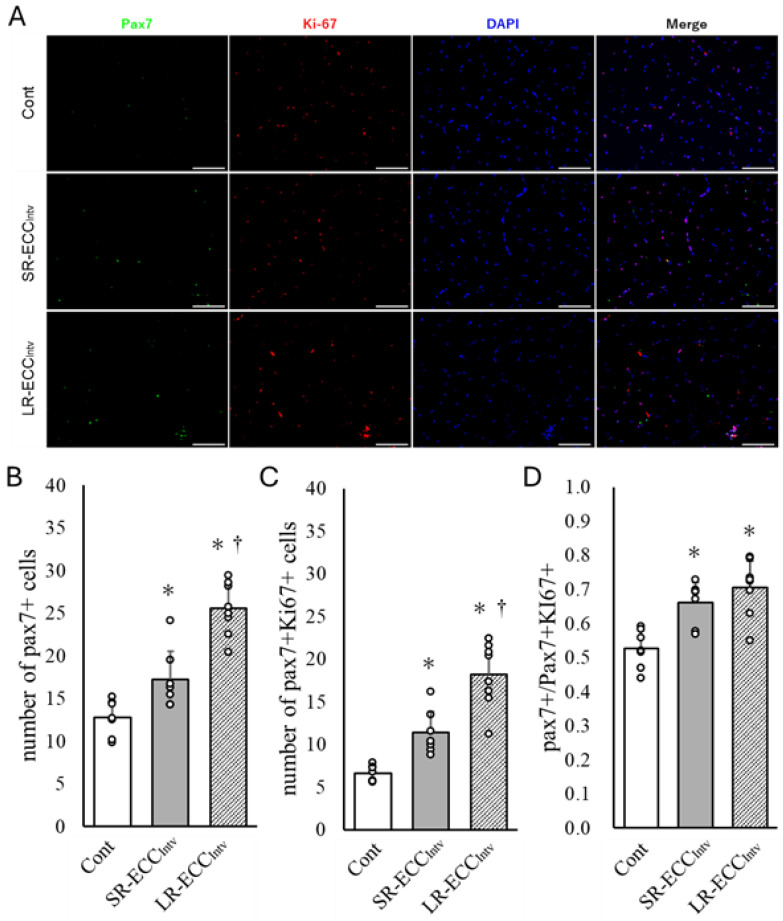
Representative cross-sectional images of tibialis anterior (TA) muscle immunostained for Pax7 (green) and Ki67 (red) in the Cont, SR-ECC_Intv_, and LR-ECC_Intv_ groups (**A**), quantification of the mean number of Pax7+ cells (**B**), the number of Pax7+ and Ki67+ double-positive cells (**C**), and the percentage of Pax7+ and Ki67 double-positive cells among Pax7+ cells (**D**). Blue indicates 4′,6-Diamidino-2-phenylindole (DAPI). The scale bar represents 100 μm. * *p* < 0.05 vs. Cont, ^†^ *p* < 0.05 vs. SR-ECC_Intv_. Values are expressed as means ± SD.

The results showed that the SR-ECC_Intv_ group showed a 35.3% increase in the number of Pax7+ cells and a 71.8% increase in Pax7+ and ki67+ cells compared to the Cont group (*p* < 0.05) (Figure 5A–C). These increases in SR-ECC_Intv_ were smaller than those in LR-ECC_Intv_ (*p* < 0.05) (Figure 5A–C). The ratio of MuSCs proliferation showed a significant increase in both SR-ECC_Intv_ and LR-ECC_Intv_ compared to the Cont group, and there was no difference between the two groups (*p* < 0.05) (Figure 5D).

## 3. Discussion

Here, we present findings on the effects of SR-ECCs on the muscle morphology and function of rat skeletal muscle. We observed that SR-ECCs avoided muscle damage and myofibrosis, promoted the proliferation of MuSCs, and resulted in changes in the distribution of FCSA. To our knowledge, this is the first study examining the effect of SR-ECC intervention without inducing muscle damage.

ECC-induced muscle damage depends on the intensity, volume, angular velocity, and muscle elongation of ECC [8]. Among these factors, the degree of muscle elongation is strongly influenced by the magnitude of muscle damage [12]. Small muscle elongation during ECC reduces the amount of muscle tissue damage, resulting in inflammation, loss of muscle strength, and muscle soreness [10,11,18,19]. In this study, the range of motion during ECC was limited to 5°, suggesting that the reduced muscle stretch contributed to the minimized muscle tissue damage observed. This finding underscores the importance of controlling muscle stretch to mitigate ECC-induced muscle damage. It should also be noted that the intensity, total ECC volume, and angular velocity were matched across both ECC protocols. These parameters are also known to be associated with muscle damage [8]. Several studies examining the effects of different ECC ranges of motion have shown that total ECC volume and/or angular velocity are not the same [10,11,18,19]. The SR-ECC used in this study was matched to the LR-ECC joint range of motion of 100° by performing 20 repetitions at a 5° joint range of motion, thereby normalizing the total ECC volume. Both ECC protocols were conducted at the same angular velocity and electrical stimulation intensity, with tetanic contractions being induced. We show that intensity, ECC volume, and angular velocity were consistent among the factors affecting muscle damage, suggesting that a small range of motion of ECC reduces muscle tissue damage. On the other hand, muscle damage was observed after the LR-ECC single-bout. However, after repeated ECC over a 4-week period, membrane permeability improved, and muscle damage was largely absent. Repeated exposure to ECC is widely recognized to provide protection against muscle damage by increasing connective tissue to withstand ECC stress (i.e., repeated bout effect) [20,21]. LR-ECC_Intv_ showed an increase in connective tissue after 4 weeks of exercise intervention. These findings suggest that while initial LR-ECC induced muscle damage, continued LR-ECC intervention protected against further damage through myofibrosis.

ECC increases membrane permeability by disrupting muscle membranes and activating SACs due to mechanical stress [22]. This leads to an influx of intracellular ions, such as calcium ions (Ca^2+^), and proteins from outside the muscle cell [22]. EBD is used as a marker to evaluate muscle membrane permeability and myofiber damage [23]. ECC increases intracellular Ca^2+^ concentrations in muscle cells with increased membrane permeability and promotes the production of calcium-dependent proteases such as calpain and phospholipase [22]. These proteases may degrade muscle proteins and disrupt the structures responsible for force production and transmission, ultimately leading to a reduction in contractility [22,24]. LR-ECCs showed many dystrophin-negative and EBD+ fibers, whereas SR-ECCs did not. These findings suggest that the muscle force loss in LR-ECCs may be caused by muscle membrane damage. This damage may lead to increased membrane permeability and disruption of muscle cell structure. Notably, SR-ECCs showed the same muscle force loss as LR-ECCs with muscle damage, despite maintaining muscle membrane integrity and permeability. This suggests that SR-ECC-induced muscle force loss may not be due to the increase in intracellular Ca^2+^ concentration associated with membrane permeability. Multiple factors contribute to ECC-induced muscle force loss [25]. Following ECC, the disruption of excitation–contraction (E-C) coupling and Z-line streaming may occur, which are likely associated with muscle force loss [26,27,28]. Despite maintaining muscle membrane integrity, SR-ECCs may lead to ultrastructure damage related to E-C coupling and sarcomere structures. However, the mechanisms of muscle force loss in SR-ECCs are unclear and require further research.

The mechanistic target of rapamycin complex 1 is a regulator of protein synthesis in muscle, activated by several factors, including insulin-like growth factor-1, insulin, AMP-activated protein kinase, and amino acids, with mechanical stress being a particularly important factor during exercise [2]. Skeletal muscle mechanosensors, including SACs and integrins in the plasma membrane, contribute to the induction of protein synthesis via mechanotransduction [2,6,17,29]. In our study, SR-ECC was performed to apply moderate mechanical stress to the membrane expressing mechanosensors while avoiding muscle tissue damage by controlling muscle elongation. We found that SR-ECC_Intv_ induced an increase in the number of fibers with a large FCSA. However, this increase was smaller than that observed with LR-ECC_Intv_. These findings suggest that the degree of muscle elongation in the designed ECC protocol may influence protein synthesis through mechanosensors localized in the muscle during chronic ECC exercise interventions.

The two main pathways promoting myonuclear proliferation are triggered by mechanical stress and inflammation during muscle regeneration [16]. In the former, mesenchymal progenitor cells within the muscle respond to mechanical stress by promoting the proliferation of MuSCs in a Yap/Taz-dependent manner [16]. Recent studies have reported that concentric contraction exercise interventions increase Pax7 and MyoD expression [15]. SR-ECC_Intv_ increased the number of MuSCs and promoted MuSC proliferation at levels comparable to LR-ECC_Intv_. It is likely that SR-ECC stimulates MuSC proliferation in response to mechanical stress through the Yap/Taz pathway without inducing inflammation typically associated with muscle damage. Moreover, numerous researchers have investigated the necessity of MuSCs in muscle hypertrophy. McCarthy et al. [30] reported that MuSC-deficient mice, following surgical removal of the synergist muscle, exhibited muscle hypertrophy comparable to that of normal mice two weeks after resection. However, Fry et al. [31] extended this loading period to eight weeks in the same MuSC-deficient mice and observed that the muscle hypertrophy response was attenuated in the absence of MuSCs. These findings outline the importance of MuSCs for long-term muscle hypertrophy. Therefore, SR-ECC exercise interventions over a longer term (< 8 weeks) may induce muscle hypertrophy more effectively, given that the number of myonuclei is increased upon MuSC proliferation. Notably, our data indicate that SR-ECC_Intv_ might have potential for inducing muscle hypertrophy without causing myofibrosis, which is defined as an increase in connective tissue within muscle tissue. Myofibrosis is caused by the activation of fibroblasts in response to inflammation and mechanical or chemical stimulation during muscle regeneration [32]. Our results suggest that SR-ECC_Intv_ provided exercise stimulation without causing myofibrosis, likely due to the lack of inflammation in SR-ECCs associated with muscle damage. Exercise-induced muscle hypertrophy without myofibrosis could serve as an effective exercise method for combating aging and immobilization-induced myofibrosis.

The present study has several limitations. First, the sample sizes between the groups are unequal and small (*n* = 4 for SR-ECC_SB_ and LR-ECC_SB_ groups, *n* = 7–8 for SR-ECC_Intv_ and LR-ECC_Intv_ groups). These small and unbalanced sample sizes may also influence the statistical power of the study, although the LR-ECC_SB_ group was significantly greater than the SR-ECC_SB_ and LR-ECC_Intv_ groups in muscle damage parameters. A larger and more balanced sample size might confirm our findings and enhance their statistical significance and generalizability. Second, we counted the total number of EBD+ myofibers in the whole muscle cross-section. Normalizing EBD+ myofibers to the total cell count would provide more insightful data. Third, the study did not examine the muscle ultrastructure, including E-C coupling, changes in Ca^2+^ levels, or biochemical indicators of contractile proteins after exercise intervention. Therefore, although SR-ECCs did not cause muscle tissue damage, the reduction in muscle contractile function occurred after chronic exercise intervention, but the factors that contributed to this reduction remain unknown. It is important to clarify the determinants of ECC-induced muscle dysfunction to expand the potential applications of ECC. Finally, we have not been able to examine the muscle hypertrophy pathway or the increase in the number of myonuclei. Clarification of these factors may establish a method for achieving high exercise effects at a low impact without the accompanying muscle damage.

In summary, our data show that short-range ECC resulted in a smaller increase in the number of fibers with a large FCSA over a 4-week exercise intervention compared to large-range ECC, without causing muscle damage and myofibrosis; furthermore, a 4-week SR-ECC intervention might promote MuSC proliferation. The relevance of the current data about the ES-induced muscle force after the 4-week ECC intervention is that both ECC interventions induced a force deficit in twitch contraction but not in tetanic contraction. Future studies should investigate muscle tissue adaptations with prolonged SR-ECC exercise.

## 4. Materials and Methods

### 4.1. Animals and Experimental Protocol

We performed two separate experiments as described below.

#### 4.1.1. Experiment 1

First, a single bout of the small-range ECC (SR-ECC) and large-range ECC (LR-ECC) protocols was performed on the tibialis anterior muscle to assess the level of muscle damage. Male Fischer 344 rats (CLEA, Tokyo, Japan) were housed under a 12 h light–dark cycle with ad libitum access to CE-2 rodent chow (CLEA, Tokyo, Japan) and water. The rats (12-week-old, *n* = 8) were randomly assigned to one of the following groups: small-range ECC single-bout (SR-ECC_SB_, *n* = 4) or large-range ECC single-bout (LR-ECC_SB_, *n* = 4). Only one bout of each ECC protocol was performed for this experiment.

#### 4.1.2. Experiment 2

We assessed the effects of exercise interventions on muscle tissue and muscle function in both SR-ECC and LR-ECC protocols. The rats (12-week-old, *n* = 22) were randomly assigned to one of the following groups: small-range ECC intervention (SR-ECC_Intv_, *n* = 7), large-range ECC intervention (LR-ECC_Intv_, *n* = 8), or control with anesthesia during the exercise intervention (Cont, *n* = 7). The SR-ECC and LR-ECC procedures were carried out as described in Experiment 1. The control group was anesthetized at the same time as the exercise intervention groups. Each exercise intervention was performed twice a week (Thursday and Sunday) over a span of four weeks.

### 4.2. ECC Procedures Induced by Electrical Stimulation

ECC was performed by transcutaneous electrical stimulation (100 Hz, 20–30 V, 2 s) of the left TA muscle using an electrical stimulator (SEN-7203, Nihon Kohden, Tokyo, Japan) and isolator (SS-201J, Nihon Kohden, Tokyo, Japan) under isoflurane anesthesia (1.5–2%) [33,34]. SR-ECC consisted of ankle plantar dorsiflexion exercises at an angular velocity of 100°/sec, with an ankle joint range of motion from 135° to 140° (i.e., a 5° range of motion). SR-ECC was defined as one exercise with 20 repetitions (Table 1). LR-ECC consisted of ankle plantar flexion exercises at an angular velocity of 100°/s and an ankle joint range of motion from 80° to 180° (i.e., a 100° range of motion) (Table 1) [11,35]. Each group performed a total of eight sets of ten exercises, consisting of two seconds of exercise followed by eight seconds of rest, with a rest period of three minutes between each set.

### 4.3. EBD Injection

Evans blue dye (EBD) binds to serum albumin and is transported into damaged muscle fibers alongside the albumin, which enables the detection of muscle fiber damage. To identify muscle fibers increased heightened membrane permeability, an intraperitoneal injection of 1% EBD solution, equivalent to 1% of the body weight, was administered 24 h prior to muscle sampling [33,34].

### 4.4. Muscle Contraction Force Measurement

The maximal isometric contraction force of the TA muscle was measured by transcutaneous electrical stimulation immediately before muscle sampling (i.e., two days after the last ECC bout) [36,37,38]. Rats were placed under isoflurane anesthesia (2–2.5%) and positioned on a working platform equipped with restraining bars and pins at the knee and ankle joints. The distal tendon of the TA muscle was aligned with the direction of muscle pull and connected to an isometric transducer (TB-654T; Nihon Kohden, Tokyo, Japan), which was securely fastened using 4-0 suture silk on a three-dimensional drive precision stage. The maximal ES intensity was determined by inducing twitches at 1 Hz and gradually increasing the intensity until maximal twitch was achieved. Subsequently, using the maximal ES intensity, the ES frequency was progressively increased to determine the frequency that produced maximal tension. The maximal isometric contraction tension was measured at least twice using each determined ES protocol, and the maximum value is representative. Signals were captured using a PowerLab A/D converter (AD Instruments, Nagoya, Japan) at a sampling frequency of 2 kHz. Maximal rates of force development (+dP/dt) and time to peak were derived from the contraction curves.

### 4.5. Tissue Preparation

Following the measurement of muscle contraction force, TA muscles were harvested and weighed. TA muscle weight was normalized to body weight. For histological analysis, the middle section of the TA muscles was trimmed and statically mounted on a piece of cork with OCT compound. The TA muscles were then immersed in isopentane cooled with liquid nitrogen for tissue embedding. Muscle samples were stored at −80 °C until use.

### 4.6. Histological Analysis

The TA muscle samples were cut into 10 µm cross-sections using a cryostat (CM3050S; Leica, Nussloch, Germany) maintained at −20 °C and were mounted on salinized slides. Transverse sections of the TA muscle belly were imaged using a fluorescence microscope (BX60; Olympus, Tokyo, Japan) equipped with a charge-coupled device (CCD) camera (DP73; Olympus, Tokyo, Japan) to determine the number of EBD-positive (+) myofibers. The number of EBD+ myofibers was quantified across the entire cross-sectional area of the TA muscle tissue. A picrosirius red (PSR) staining kit (Polysciences, Inc., Warrington, PA, USA) was used to visualize the connective tissue within the muscle tissue. TA muscle cross-sections were fixed with 4% paraformaldehyde (PFA), rinsed in distilled water, and incubated with PSR solution for 5 min at room temperature. Subsequently, the sections were treated with 0.1 M HCl, rinsed in distilled water, dehydrated in ethanol, permeabilized with xylene, and mounted.

For immunohistochemical analysis, TA muscle cross-sections were fixed with 4% PFA, washed with 0.1 M phosphate-buffered saline (PBS), and blocked with a mixture of 1% Triton X-100, 10% normal goat serum (NGS), and 0.1 M PBS for 1 h at room temperature, and then washed twice with PBS for 5 min. The TA muscle was treated with the following primary antibodies: mouse monoclonal anti-dystrophin antibody (D8168; 1:250; Merck), mouse anti-Pax7 antibody (MAB1675; 1:200; Funakoshi, a satellite cell marker), and rabbit anti-Ki67 antibody (ab15580, 1:200; Abcam, a cell proliferation marker). After primary antibody treatment, the sections were incubated with 0.3% Triton X-100 in PBS for 16–20 h at 4 °C. Next, the sections were washed several times with 0.1 M PBS and incubated with the appropriate secondary antibodies (Alexa Fluor 488-conjugated goat anti-mouse IgG antibody, 1:500, ab150117, Abcam; Alexa Fluor 568-conjugated goat anti-rabbit IgG antibody, 1:500, ab175471, Abcam) diluted in PBS containing 2.5% NGS and 1% Triton X-100 at room temperature for 1 h. Following several washes with PBS, the samples were sealed with a mounting medium containing 4′,6-diamidino-2-phenylindole (DAPI).

Immunofluorescent and PSR-stained images of TA muscle sections were captured with a light/fluorescence microscope (BZ-X710, Keyence, Osaka, Japan). The number of EBD+ myofibers was counted in the whole muscle cross-section. The PSR+ mean cross-sectional area ratio was calculated from five randomly selected microscope fields. The myofiber cross-sectional area (FCSA) of at least 150 fibers in each TA muscle was measured from fluorescence microscopic images [39]. The numbers of MuSCs and proliferating MuSCs were determined as the averages of the counts of 10 random microscopic fields. All image analyses were performed using image analysis software (Image-Pro Premier 9.1; Media Cybernetics, Rockville, MD, USA).

### 4.7. Statistical Analysis

Statistical analyses were performed using the SPSS Statistical Package Version 25.0 (IBM, Chicago, IL, USA). The data are presented as mean ± standard deviation. Differences between groups were assessed using a one-way analysis of variance (ANOVA) followed by Tukey’s post hoc test. The two-sample Kolmogorov–Smirnov test was used to compare the frequency distribution of the myofiber cross-sectional area. Statistical significance was set at *p* < 0.05.

## Figures and Tables

**Table 1:** Parameters for SR-ECC and LR-ECC exercise protocols.

Parameters	SR-ECC	LR-ECC
Range of motion (°)	5	100
Angular velocity (°/s)	100	100
Repetitions per exercise (reps/exercise)	20	1
Number of exercises (exercises/set)	10	10
Sets (sets)	8	8

SR-ECC = 8 sets × 10 exercises/set × 20 reps/exercise × 5 degrees = 8000 degrees in total, LR-ECC = 8 sets × 10 exercises/set × 1 rep/exercise × 100 degrees = 8000 degrees in total.

## Data Availability

The original contributions presented in the study are included in the article material; further inquiries can be directed to the corresponding author.

## References

[1] LaStayo P., Marcus R., Dibble L., Frajacomo F., Lindstedt S. (2014). Eccentric exercise in rehabilitation: Safety, feasibility, and application. J. Appl. Physiol..

[2] Roberts M.D., McCarthy J.J., Hornberger T.A., Phillips S.M., Mackey A.L., Nader G.A., Boppart M.D., Kavazis A.N., Reidy P.T., Ogasawara R. (2023). Mechanisms of mechanical overload-induced skeletal muscle hypertrophy: Current understanding and future directions. Physiol. Rev..

[3] Herzog W. (2014). Mechanisms of enhanced force production in lengthening (eccentric) muscle contractions. J. Appl. Physiol..

[4] Nishikawa K.C., Monroy J.A., Uyeno T.E., Yeo S.H., Pai D.K., Lindstedt S.L. (2012). Is titin a ‘winding filament’? A new twist on muscle contraction. Proc. Biol. Sci..

[5] Guharay F., Sachs F. (1984). Stretch-activated single ion channel currents in tissue-cultured embryonic chick skeletal muscle. J. Physiol..

[6] Spangenburg E.E., McBride T.A. (2006). Inhibition of stretch-activated channels during eccentric muscle contraction attenuates p70S6K activation. J. Appl. Physiol..

[7] McHugh M.P. (2003). Recent advances in the understanding of the repeated bout effect: The protective effect against muscle damage from a single bout of eccentric exercise. Scand. J. Med. Sci. Sports.

[8] Nosaka K., Aldayel A., Jubeau M., Chen T.C. (2011). Muscle damage induced by electrical stimulation. Eur. J. Appl. Physiol..

[9] Brooks S.V., Zerba E., Faulkner J.A. (1995). Injury to muscle fibres after single stretches of passive and maximally stimulated muscles in mice. J. Physiol..

[10] Fochi A.G., Damas F., Berton R., Alvarez I., Miquelini M., Salvini T.F., Libardi C.A. (2016). Greater eccentric exercise-induced muscle damage by large versus small range of motion with the same end-point. Biol. Sport.

[11] Hayashi K., Katanosaka K., Abe M., Yamanaka A., Nosaka K., Mizumura K., Taguchi T. (2017). Muscular mechanical hyperalgesia after lengthening contractions in rats depends on stretch velocity and range of motion. Eur. J. Pain.

[12] Talbot J.A., Morgan D.L. (1998). The effects of stretch parameters on eccentric exercise-induced damage to toad skeletal muscle. J. Muscle Res. Cell Motil..

[13] Cermak N.M., Snijders T., McKay B.R., Parise G., Verdijk L.B., Tarnopolsky M.A., Gibala M.J., Van Loon L.J. (2013). Eccentric exercise increases satellite cell content in type II muscle fibers. Med. Sci. Sports Exerc..

[14] Hyldahl R.D., Olson T., Welling T., Groscost L., Parcell A.C. (2014). Satellite cell activity is differentially affected by contraction mode in human muscle following a work-matched bout of exercise. Front. Physiol..

[15] Karimi Majd S., Gholami M., Bazgir B. (2023). PAX7 and MyoD Proteins Expression in Response to Eccentric and Concentric Resistance Exercise in Active Young Men. Cell J..

[16] Fukada S.I., Higashimoto T., Kaneshige A. (2022). Differences in muscle satellite cell dynamics during muscle hypertrophy and regeneration. Skelet. Muscle.

[17] Boppart M.D., Mahmassani Z.S. (2019). Integrin signaling: Linking mechanical stimulation to skeletal muscle hypertrophy. Am. J. Physiol. Cell Physiol..

[18] Baroni B.M., Pompermayer M.G., Cini A., Peruzzolo A.S., Radaelli R., Brusco C.M., Pinto R.S. (2017). Full Range of Motion Induces Greater Muscle Damage Than Partial Range of Motion in Elbow Flexion Exercise With Free Weights. J. Strength Cond. Res..

[19] Váczi M., Costa A., Rácz L., Tihanyi J. (2009). Effects of consecutive eccentric training at different range of motion on muscle damage and recovery. Acta Physiol. Hung..

[20] Hyldahl R.D., Chen T.C., Nosaka K. (2017). Mechanisms and Mediators of the Skeletal Muscle Repeated Bout Effect. Exerc. Sport Sci. Rev..

[21] McHugh M.P., Connolly D.A., Eston R.G., Gleim G.W. (1999). Exercise-induced muscle damage and potential mechanisms for the repeated bout effect. Sports Med..

[22] Allen D.G., Whitehead N.P., Yeung E.W. (2005). Mechanisms of stretch-induced muscle damage in normal and dystrophic muscle: Role of ionic changes. J. Physiol..

[23] Hamer P.W., McGeachie J.M., Davies M.J., Grounds M.D. (2002). Evans Blue Dye as an in vivo marker of myofibre damage: Optimising parameters for detecting initial myofibre membrane permeability. J. Anat..

[24] Duncan C.J. (1978). Role of intracellular calcium in promoting muscle damage: A strategy for controlling the dystrophic condition. Experientia.

[25] Warren G.L., Ingalls C.P., Lowe D.A., Armstrong R.B. (2001). Excitation-contraction uncoupling: Major role in contraction-induced muscle injury. Exerc. Sport Sci. Rev..

[26] Balnave C.D., Allen D.G. (1995). Intracellular calcium and force in single mouse muscle fibres following repeated contractions with stretch. J. Physiol..

[27] Chen W., Ruell P.A., Ghoddusi M., Kee A., Hardeman E.C., Hoffman K.M., Thompson M.W. (2007). Ultrastructural changes and sarcoplasmic reticulum Ca2+ regulation in red vastus muscle following eccentric exercise in the rat. Exp. Physiol..

[28] Yasuda T., Sakamoto K., Nosaka K., Wada M., Katsuta S. (1997). Loss of sarcoplasmic reticulum membrane integrity after eccentric contractions. Acta Physiol. Scand..

[29] Schoenfeld B.J., Wackerhage H., De Souza E. (2022). Inter-set stretch: A potential time-efficient strategy for enhancing skeletal muscle adaptations. Front. Sports Act. Living.

[30] McCarthy J.J., Mula J., Miyazaki M., Erfani R., Garrison K., Farooqui A.B., Srikuea R., Lawson B.A., Grimes B., Keller C. (2011). Effective fiber hypertrophy in satellite cell-depleted skeletal muscle. Development.

[31] Fry C.S., Lee J.D., Jackson J.R., Kirby T.J., Stasko S.A., Liu H., Dupont-Versteegden E.E., McCarthy J.J., Peterson C.A. (2014). Regulation of the muscle fiber microenvironment by activated satellite cells during hypertrophy. FASEB J..

[32] Mahdy M.A.A. (2019). Skeletal muscle fibrosis: An overview. Cell Tissue Res..

[33] Hayao K., Tamaki H., Nakagawa K., Tamakoshi K., Takahashi H., Yotani K., Ogita F., Yamamoto N., Onishi H. (2018). Effects of Streptomycin Administration on Increases in Skeletal Muscle Fiber Permeability and Size Following Eccentric Muscle Contractions. Anat. Rec..

[34] Hayao K., Tamaki H., Tamakoshi K., Takahashi H., Onishi H. (2020). Myofiber Permeability and Force Production of Rat Muscles Following Eccentric Contractions: The Repeated Bout Effect Depends on the Interval. J. Biomed. Sci. Eng..

[35] Wang X.D., Kawano F., Matsuoka Y., Fukunaga K., Terada M., Sudoh M., Ishihara A., Ohira Y. (2006). Mechanical load-dependent regulation of satellite cell and fiber size in rat soleus muscle. Am. J. Physiol. Cell Physiol..

[36] Tamaki H., Tomori K., Yotani K., Ogita F., Sugawara K., Kirimto H., Onishi H., Yamamoto N., Kasuga N. (2014). Electrical stimulation of denervated rat skeletal muscle retards trabecular bone loss in early stages of disuse musculoskeletal atrophy. J. Musculoskelet. Neuronal Interact..

[37] Tamaki H., Yotani K., Ogita F., Hayao K., Kirimto H., Onishi H., Kasuga N., Yamamoto N. (2019). Low-Frequency Electrical Stimulation of Denervated Skeletal Muscle Retards Muscle and Trabecular Bone Loss in Aged Rats. Int. J. Med. Sci..

[38] Tamaki H., Yotani K., Ogita F., Sugawara K., Kirimto H., Onishi H., Kasuga N., Yamamoto N. (2015). Effect of electrical stimulation-induced muscle force and streptomycin treatment on muscle and trabecular bone mass in early-stage disuse musculoskeletal atrophy. J. Musculoskelet. Neuronal Interact..

[39] Ceglia L., Niramitmahapanya S., Price L.L., Harris S.S., Fielding R.A., Dawson-Hughes B. (2013). An evaluation of the reliability of muscle fiber cross-sectional area and fiber number measurements in rat skeletal muscle. Biol. Proced. Online.

